# Supplementary material for: Distinct genomic contexts predict gene presence-absence variation in different pathotypes of a fungal plant pathogen
Source: bioRxiv. 2023 Feb 17:2023.02.17.529015. Preprint. [Version 1] doi: 10.1101/2023.02.17.529015 (PMC9949116; doi:10.1101/2023.02.17.529015)
Supplement: 13 — Additional File 1: Supplementary Figures. Fig. S1. Phylogeny of rice-infecting M. oryzae isolates used in this study. Fig. S2. Phylogeny of wheat-infecting M. oryzae isolates used in this study. Fig. S3. Distances to the nearest gene for PAV and conserved genes in MoO and MoT. A. Density plots showing the distribution of the distances to the nearest gene for conserved and PAV genes in MoO and MoT. Fig. S4. Profile plots showing transposable element (TE) and gene density within genomic regions of the rice and wheat-infecting M. oryzae genomes. Fig. S5. Density plots of additional features of PAV and conserved genes. Fig. S6. Comparison of various functional annotations of PAV and conserved genes. Fig. S7. Density plots showing the distributions of various features of MoO and MoT genomic deletions. Fig. S8. Correlation coefficients for variables included in the MoO random forest classifier. Fig. S9. Dependence matrix of variables included in the MoO random forest classifier. Fig. S10. Confusion matrices for the MoT random forest classifier and the MoO random forest classifier trained on a subset of features. [file NIHPP2023.02.17.529015v1-supplement-13.pdf]

bioRxiv preprint doi: https://doi.org/10.1101/2023.02.17.529015; this version posted February 17, 2023. The copyright holder for this preprint (which was not certified by peer review) is the author/funder, who has granted bioRxiv a license to display the preprint in perpetuity. It is made available under aCC-BY 4.0 International license.

Additional Files

Additional File 1: Supplementary Figures.

bioRxiv preprint doi: https://doi.org/10.1101/2023.02.17.529015; this version posted February 17, 2023. The copyright holder for this preprint (which was not certified by peer review) is the author/funder, who has granted bioRxiv a license to display the preprint in perpetuity. It is made available under aCC-BY 4.0 International license.

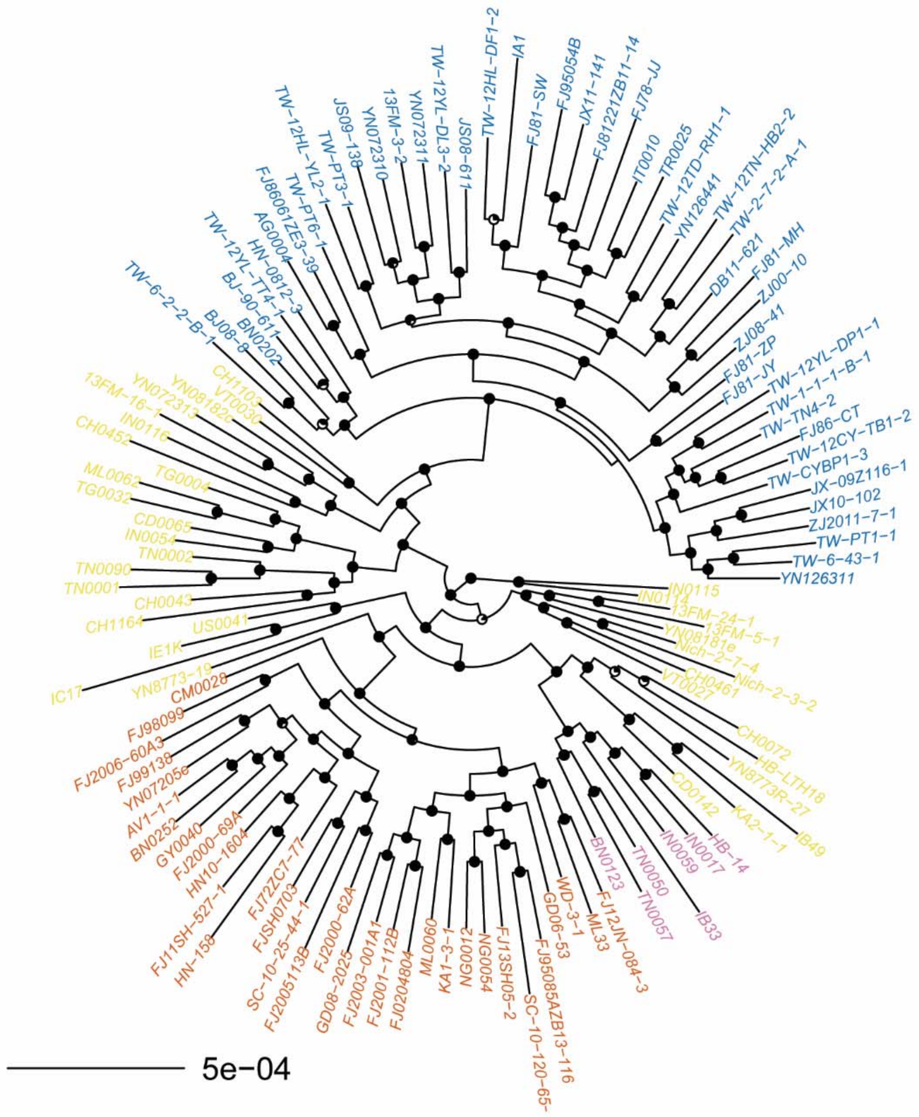

Fig. S1. Phylogeny of rice-infecting *M. oryzae* isolates used in this study. Phylogeny was generated using a multiple-sequence alignment of SCOs and fasttree [30]. Pie charts on nodes represent the fraction of bootstrap replicates that support the node. Isolates belonging to lineage 1 are colored yellow, isolates belonging to lineage 2 are colored orange, isolates belonging to lineage 3 are colored blue, and isolates belonging to lineage 4 are colored pink. Lineages were named as previously described [17].

bioRxiv preprint doi: https://doi.org/10.1101/2023.02.17.529015; this version posted February 17, 2023. The copyright holder for this preprint (which was not certified by peer review) is the author/funder, who has granted bioRxiv a license to display the preprint in perpetuity. It is made available under aCC-BY 4.0 International license.

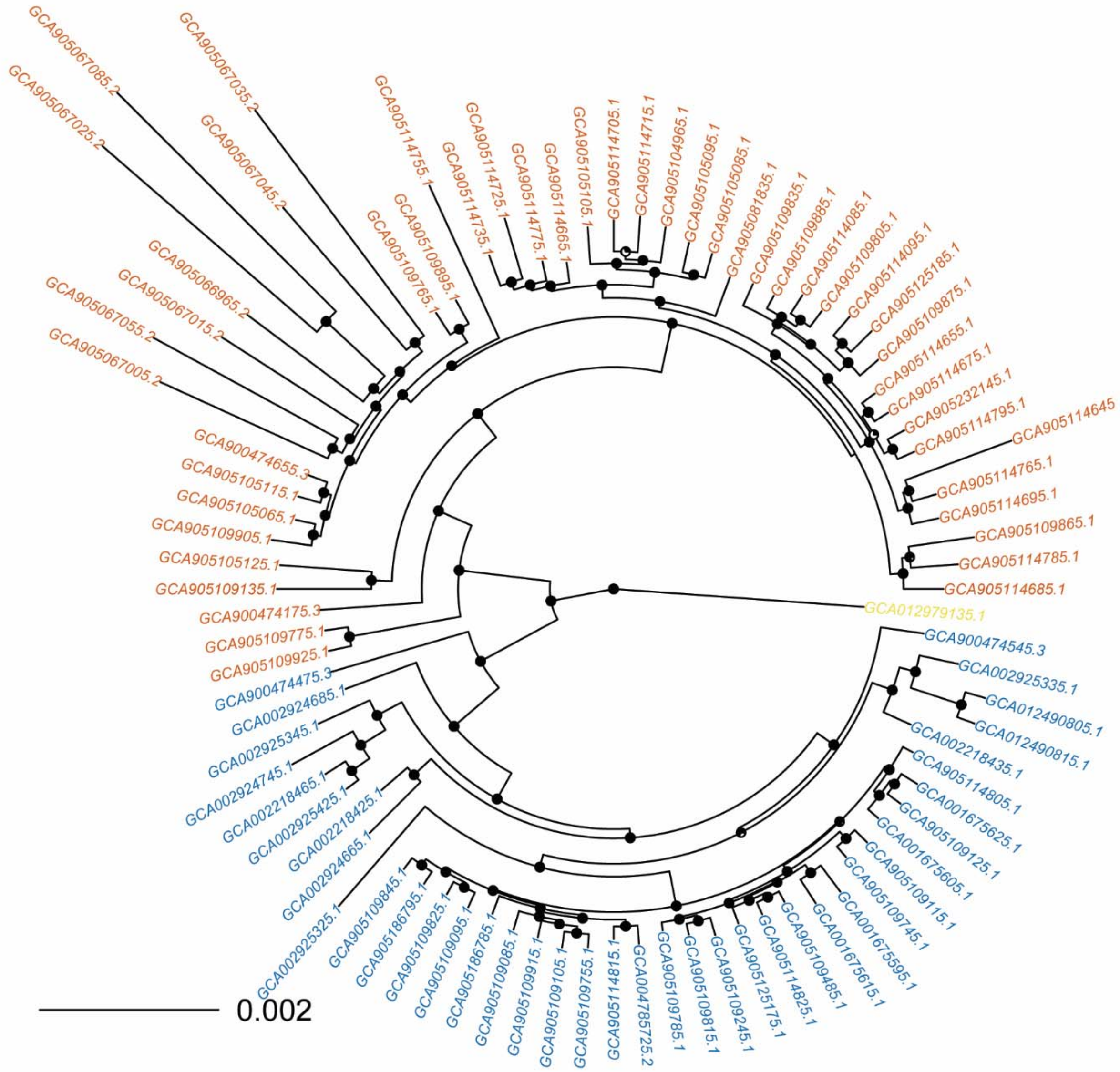

Fig. S2. Phylogeny of wheat-infecting *M. oryzae* isolates used in this study. Phylogeny was generated using a multiple-sequence alignment of SCOs and fasttree [30]. Pie charts on nodes represent the fraction of bootstrap replicates that support the node. Isolates belonging to lineage 1 are colored orange, isolates belonging to lineage 2 are colored blue, and the isolate that was not assigned to either lineage is colored yellow.

bioRxiv preprint doi: https://doi.org/10.1101/2023.02.17.529015; this version posted February 17, 2023. The copyright holder for this preprint (which was not certified by peer review) is the author/funder, who has granted bioRxiv a license to display the preprint in perpetuity. It is made available under aCC-BY 4.0 International license.

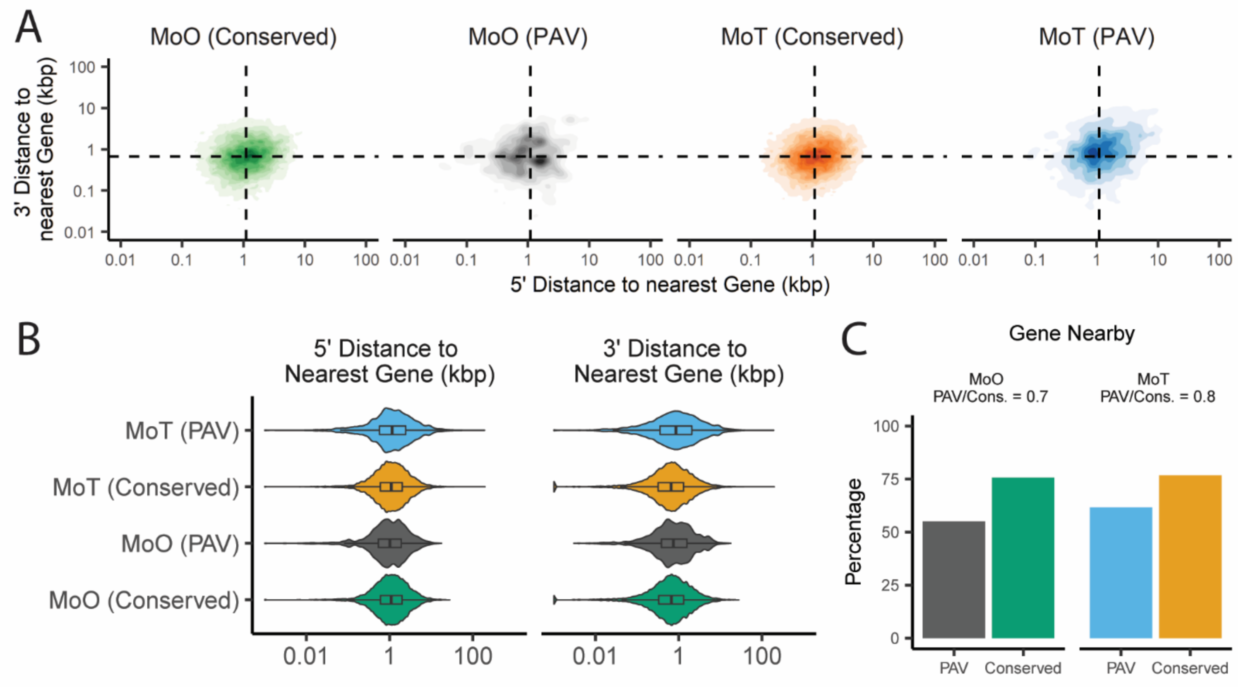

Fig. S3. Distances to the nearest gene for PAV and conserved genes in MoO and MoT. A. Density plots showing the distribution of the distances to the nearest gene for conserved and PAV genes in MoO and MoT. B. Violin plot showing the distribution of the distances to the nearest gene for conserved and PAV genes in MoO and MoT. C. Percentages and proportions of PAV and conserved genes that are within 1000bp of another gene in MoO and MoT. Dashed lines in density plots represent the median values for all genes in both pathotypes. Median values and statistical comparisons for data shown are listed in Additional File 7, Additional File 8, and Additional File 9.

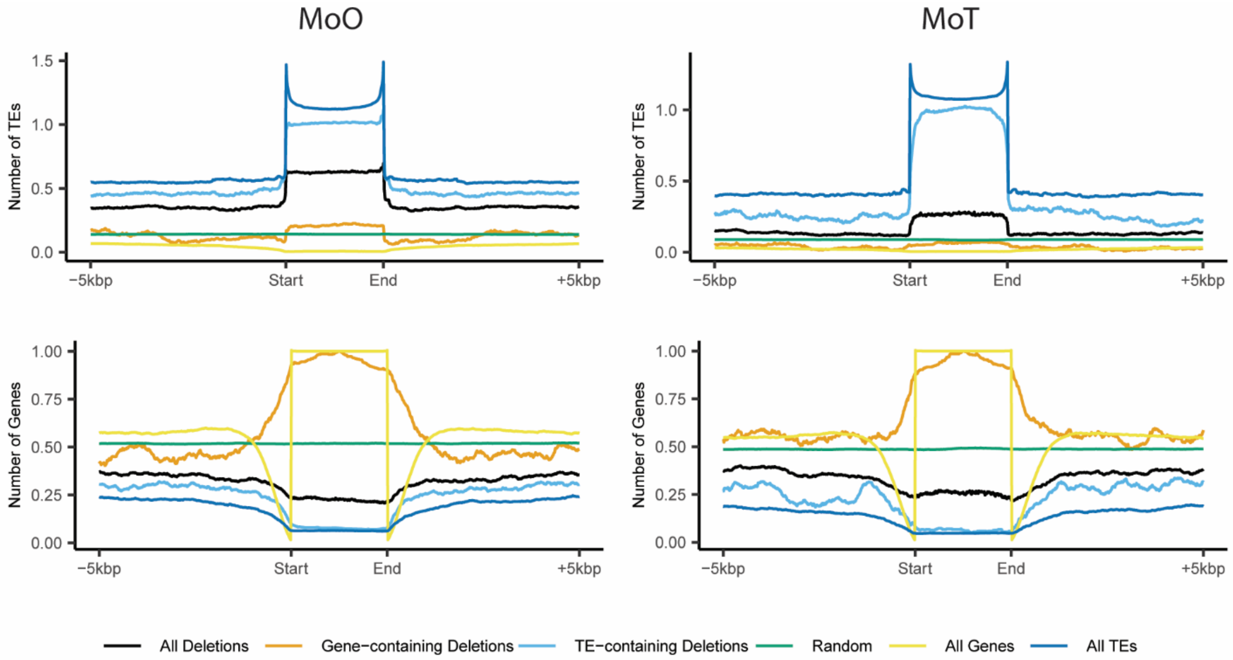

bioRxiv preprint doi: https://doi.org/10.1101/2023.02.17.529015; this version posted February 17, 2023. The copyright holder for this preprint (which was not certified by peer review) is the author/funder, who has granted bioRxiv a license to display the preprint in perpetuity. It is made available under aCC-BY 4.0 International license.

Fig. S4. Profile plots showing transposable element (TE) and gene density within genomic regions of the rice and wheat-infecting *M. oryzae* genomes. The flanking regions of these regions are also shown. Gene- and TE-containing regions represent the subet of all deletions that overlapped at least 50% with a gene or TE sequence, respectively. Genomic deletions were shuffled throughout the genome 100 times to generate the data for random regions in the plots.

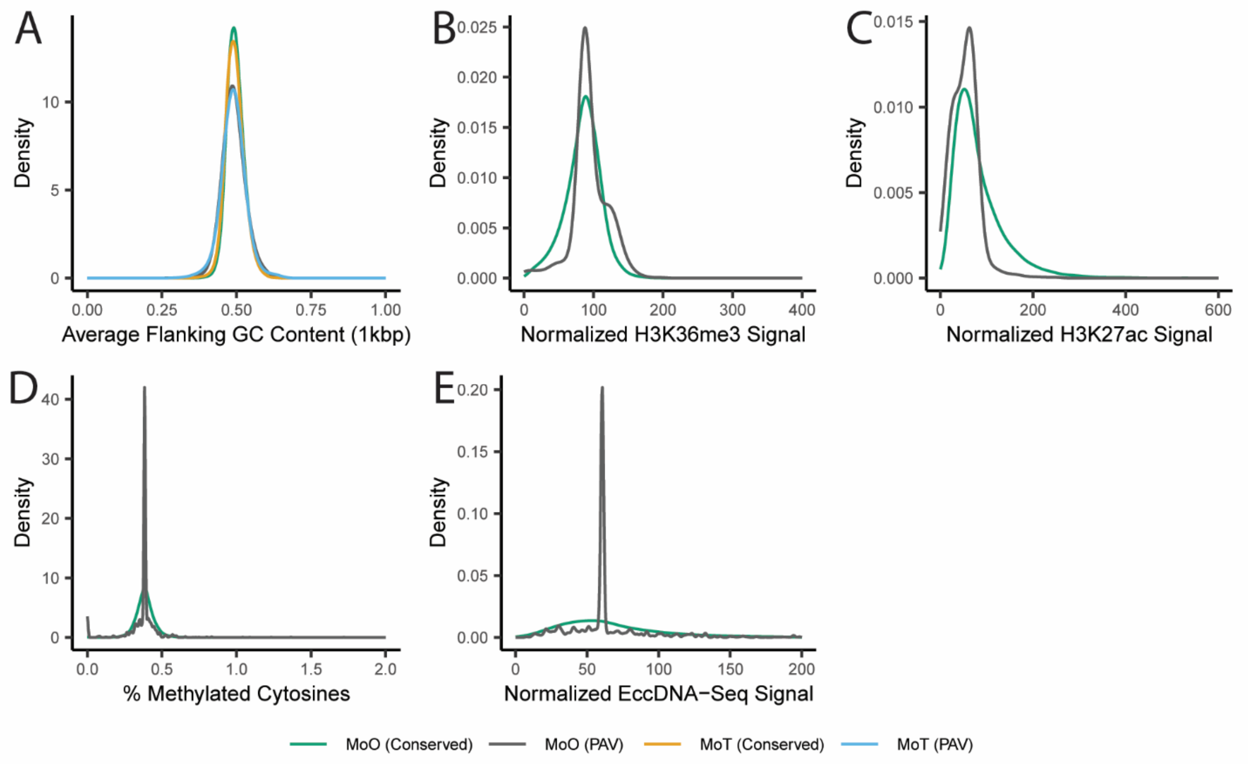

Fig. S5. Density plots of additional features of PAV and conserved genes. Density plots showing the distributions of A. average flanking GC content, B. normalized H3K36me3 histone mark ChIP-Seq signal, C. noramlized H3K27ac histone mark ChIP-Seq signal, D. average % methylation of cytosines, and E. normalized extrachromosomal DNA (eccDNA) sequencing signal for PAV and conserved genes in MoO and MoT. In panel A, the line representing the data for MoO PAV genes appears behind the line representing data for MoT PAV genes. In panels B, C, D, and E, MoT genes were not included as this data is not available for MoT. Statistics describing distributions and statistical comparisons between these statistics are listed in Additional File 10 and Additional File 11.

bioRxiv preprint doi: https://doi.org/10.1101/2023.02.17.529015; this version posted February 17, 2023. The copyright holder for this preprint (which was not certified by peer review) is the author/funder, who has granted bioRxiv a license to display the preprint in perpetuity. It is made available under aCC-BY 4.0 International license.

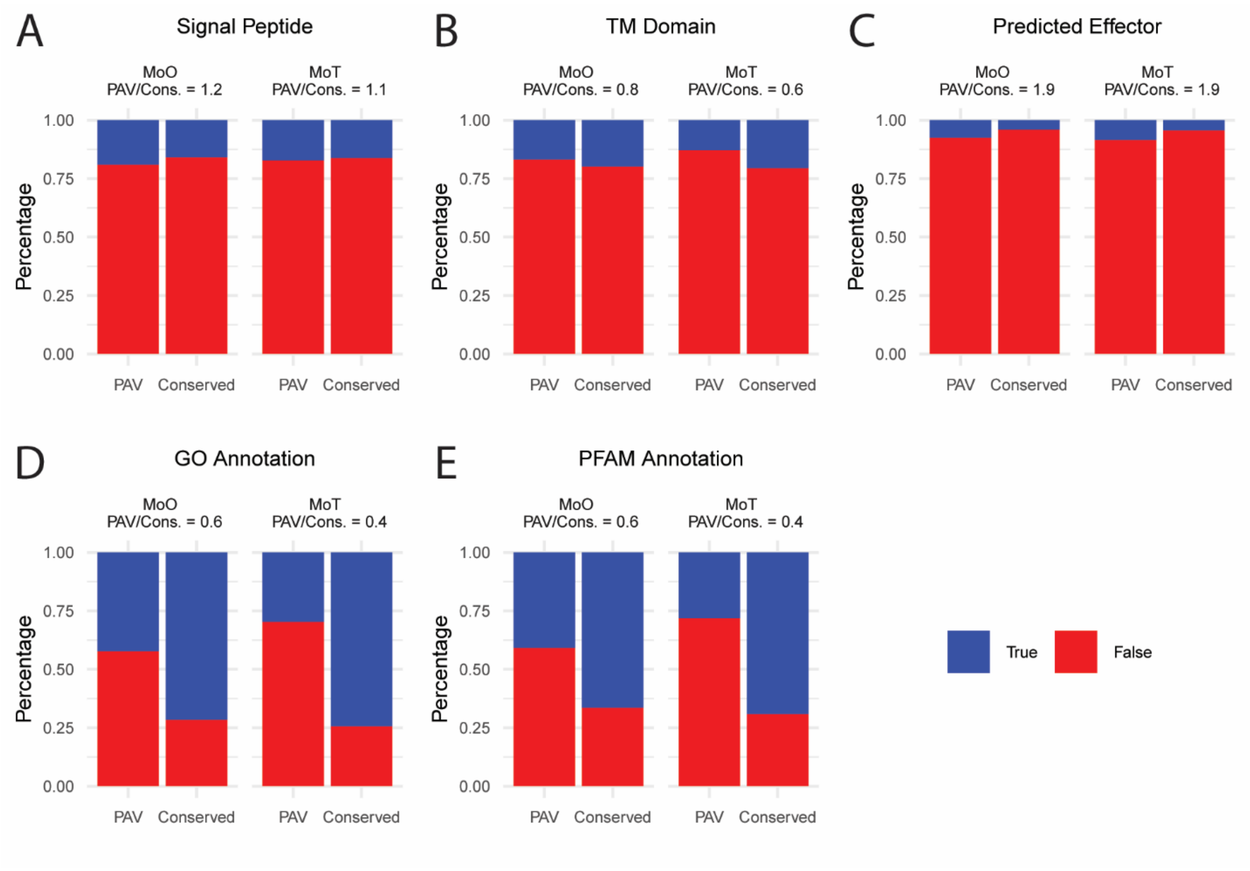

Fig. S6. Comparison of various functional annotations of PAV and conserved genes. Comparison of percentages and ratios of PAV and conserved genes annotated as A. having a signal peptide, B. having a transmembrane (TM) domain, C. being a predicted effector, D. having a GO annotation, and E. having a protein family (PFAM) domain annotation for MoO and MoT genes. Counts for each category and stastical comparisons of these counts are listed in Additional File 8 and Additional File 9.

bioRxiv preprint doi: https://doi.org/10.1101/2023.02.17.529015; this version posted February 17, 2023. The copyright holder for this preprint (which was not certified by peer review) is the author/funder, who has granted bioRxiv a license to display the preprint in perpetuity. It is made available under aCC-BY 4.0 International license.

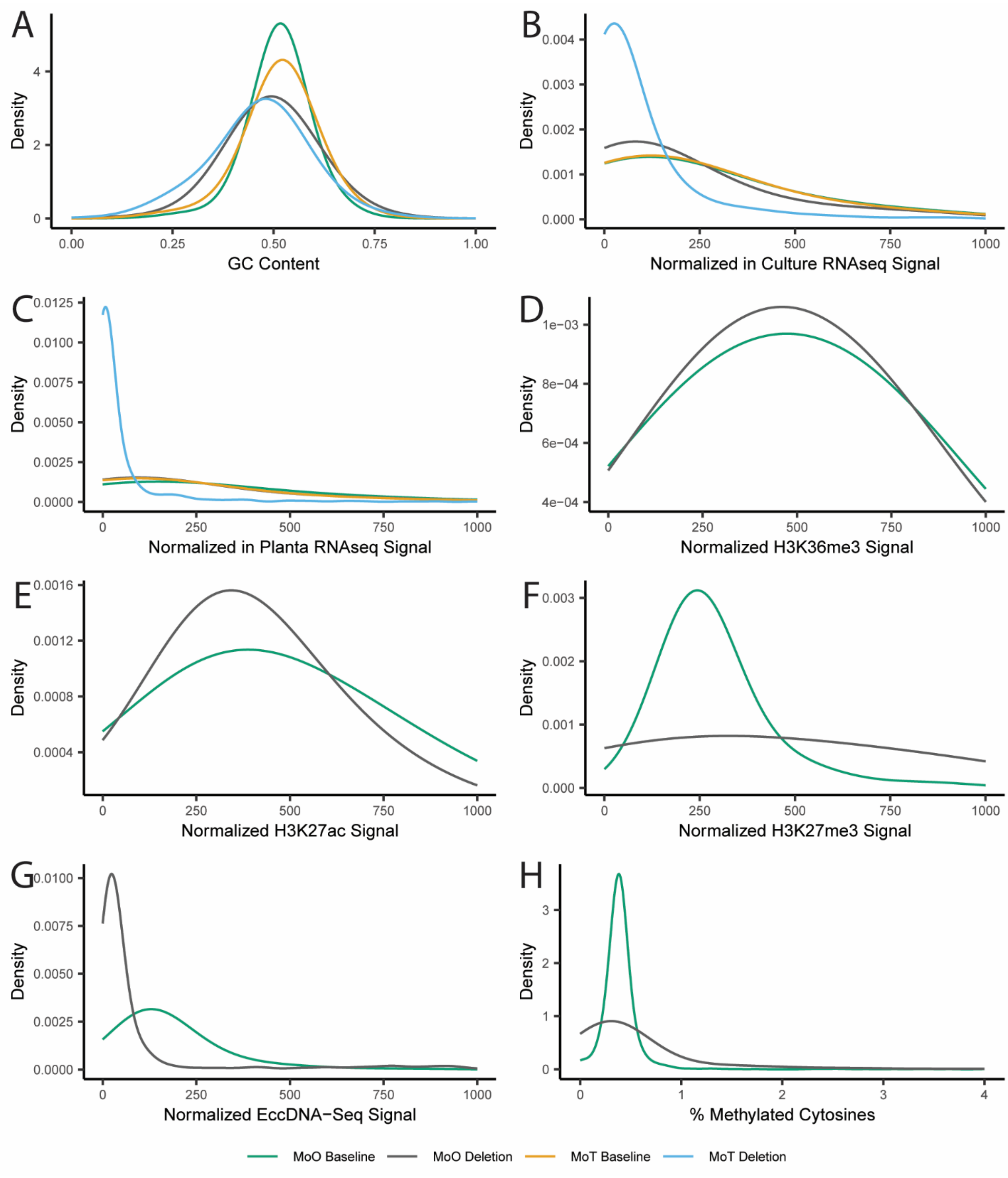

Fig. S7. Density plots showing the distributions of various features of MoO and MoT genomic deletions. Density plots showing the distributions of A. AT content, B. normalized in culture RNAseq signal, C. normalized in planta RNAseq signal, D. normalized H3K36me3 histone mark ChIP-Seq signal, E. normalized H3K27ac histone mark ChIP-Seq signal, F. normalized H3K27ac histone mark ChIP-Seq signal, G. normalized eccDNA sequencing signal, and H. average % methylation of cytosines for genomic deletions in MoO and MoT, as compared to baseline. Genomic baseline values were generated by

bioRxiv preprint doi: https://doi.org/10.1101/2023.02.17.529015; this version posted February 17, 2023. The copyright holder for this preprint (which was not certified by peer review) is the author/funder, who has granted bioRxiv a license to display the preprint in perpetuity. It is made available under aCC-BY 4.0 International license.

shuffling the deletions throughout the portions of the genome that were not deleted in any isolate. Statistics describing distributions and statistical comparisons between these statistics are listed in Additional File 12.

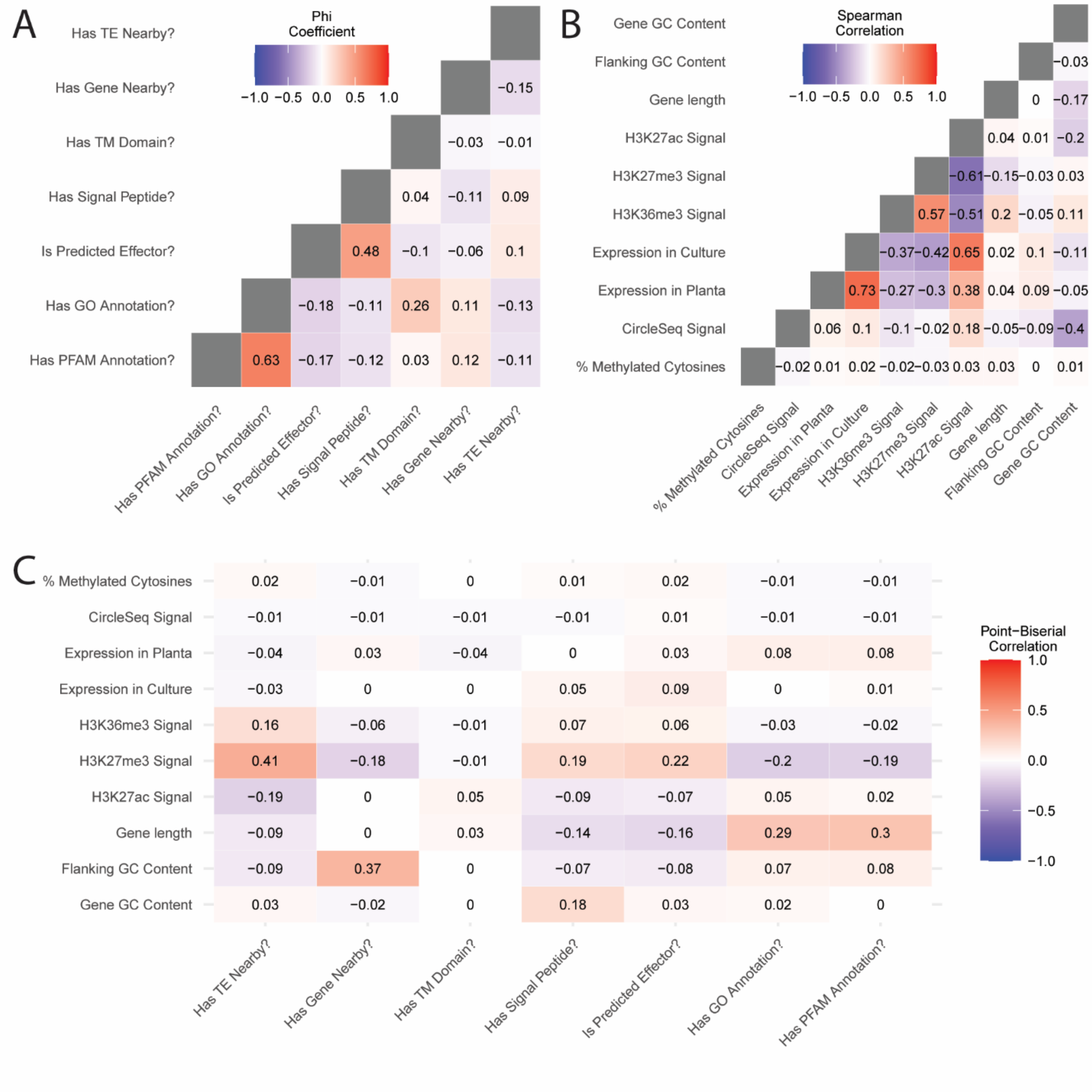

Fig. S8. Correlation coefficients for variables included in the MoO random forest classifier. Heat map representing A. Phi coefficient between binary variables, B. Spearman rank correlation coefficient between continuous variables, and C. Point-Biserial correlation coefficient between continuous and binary variables.

bioRxiv preprint doi: https://doi.org/10.1101/2023.02.17.529015; this version posted February 17, 2023. The copyright holder for this preprint (which was not certified by peer review) is the author/funder, who has granted bioRxiv a license to display the preprint in perpetuity. It is made available under aCC-BY 4.0 International license.

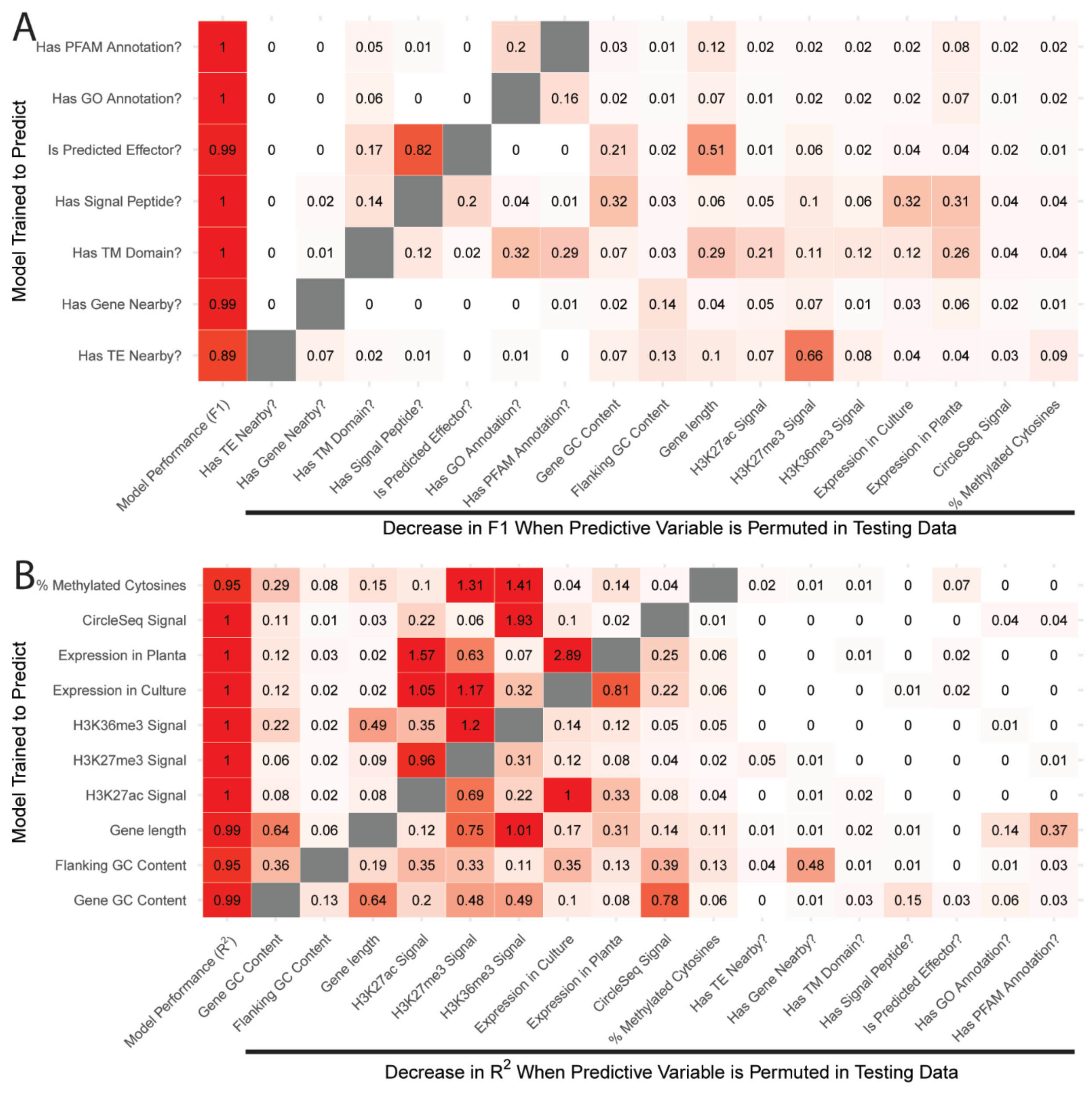

Fig. S9. Dependence matrix of variables included in the MoO random forest classifier. A model was trained to predict each variable used in our MoO random forest classifier using the remaining variables. A. Heatmap representing the F1 statistic of each model when trained to predict categorical variables and decrease in F1 when predictive variables were permuted in the testing data. B. Heatmap representing the R^2 statistic of each model when trained to predict categorical variables and decrease in R^2 when predictive variables were permuted in the testing data.

bioRxiv preprint doi: https://doi.org/10.1101/2023.02.17.529015; this version posted February 17, 2023. The copyright holder for this preprint (which was not certified by peer review) is the author/funder, who has granted bioRxiv a license to display the preprint in perpetuity. It is made available under aCC-BY 4.0 International license.

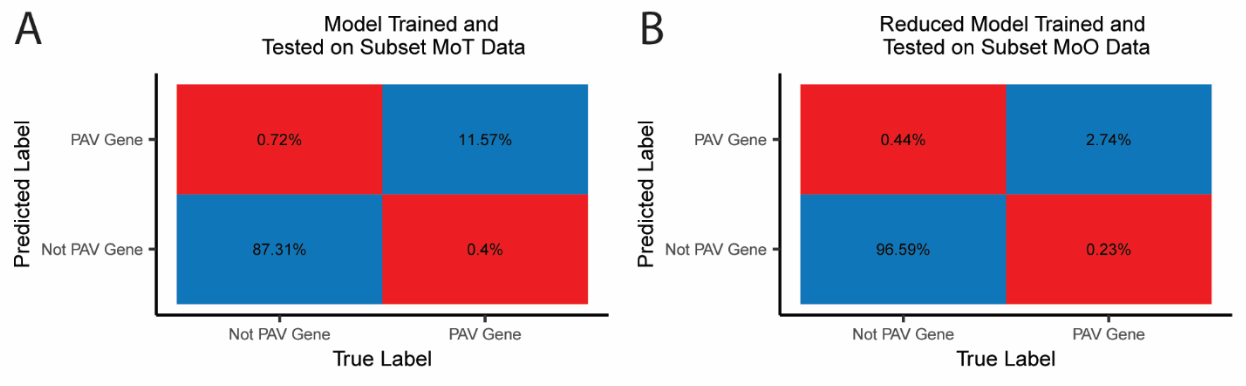

Fig. S10. Confusion matrices for the MoT random forest classifier and the MoO random forest classifier trained on a subset of features. A. Confusion matrix showing predictions of the MoT random forest classifier when tested on MoT genes that it was not trained on. B. Confusion matrix showing predictions of the MoO random forest classifier trained on a subset of features when tested on MoO genes that it was not trained on.
